# Reactive resistance to anti-angiogenic drugs

**DOI:** 10.18632/aging.100748

**Published:** 2015-05-15

**Authors:** Guilhem Bousquet, Anne Janin

**Affiliations:** Université Paris Diderot, Sorbonne Paris Cité, Laboratoire de Pathologie, UMR-S 1165, F-75010, Paris, France

Anti-angiogenic drugs have proved to be efficient in most types of cancers, and particularly in metastatic renal cell carcinoma, with several drugs currently approved for daily clinical practice. However, secondary resistance to this family of drugs is constantly observed, and the mechanisms of resistance remain poorly understood in patients.

Recently, we demonstrated that sunitinib, a tyrosine kinase inhibitor, was able to generate resistance to its own therapeutic effect *via* induced hypoxia in cancer stem cells [1].

We first analyzed human tumor samples obtained before and after treatment with sunitinib from the same patients with metastatic renal cell carcinoma. We observed that renal cancer stem-cells increased in numbers after treatment. We then reproduced this effect of sunitinib in xenografts derived from human tumor samples of metastatic renal cell carcinoma, and showed that the number of renal cancer stem-cells was directly linked to the percentage of necrosis in the tumor, either spontaneous or sunitinib-induced. In pre-clinical studies, sunitinib mainly targets neo-angiogenic microvessels, inducing necrosis. In clinical settings, there is also radiological evidence of necrosis induced by anti-angiogenic drugs in patients with metastatic renal cell carcinoma. As necrosis is an indirect marker of hypoxia, we decided to study the *in vitro* effect of experimental hypoxia on renal cancer stem-cells sorted from these renal cell carcinoma xenografts. We demonstrated that experimental hypoxia increased their resistance to sunitinib.

Our results are in accordance with the clinical observation that initial control of the tumor with sunitinib treatment is constantly followed by tumor re-growth after a median time of 11 months [2]. This is also in accordance with a recent molecular classification of renal cell carcinoma which identified two sub-types associated with primary resistance to sunitinib in patients, these sub-types being characterized by an activation of hypoxia pathways and a stem-cell signature [3].

Sunitinib, by way of its main effect on endothelial tumor cells, increases the number of renal cancer stem-cells and could thus contribute to its own resistance. Since neo-angiogenesis is a pathological process common to all tumors, efficiently targeting tumor vessels could lead to an increase in numbers of cancer stem cells, whatever the tumor type.

This opens perspectives for innovative therapeutic strategies to overcome acquired resistance to anti-angiogenic drugs:

- Should we adjust protocols on the basis of the moment of onset of necrosis in the tumor? A sequential combination of an efficient anti-angiogenic treatment followed by focal destruction of a partially necrotic residual metastasis before re-growth occurs (surgery, radiotherapy, cryoablation, radiofrequency) could easily be implemented in clinical practice.
- Another innovative therapeutic perspective might be the use of hyperoxia to decrease the number of cancer stem-cells in the resistant metastases and resensitize them.
  *In vitro*, hyperoxia restores sensitivity to drugs in chemoresistant glioblastoma cells [4]. In a pre-clinical rat model of breast carcinoma, hyperbaric oxygen treatment induced mesenchymal-to-epithelial transition of tumor cells [5], probably restoring a more differentiated phenotype.
- Why not directly target cancer stem-cells?

A major difficulty in targeting cancer stem-cells is the absence of specificity of surface markers, and thus a major risk of adverse events from direct targeting of normal cells. Even though most current data are *in vitro* and *in vivo* pre-clinical data, there are some interesting preliminary results obtained in patients with malignant tumors. By selective targeting of leukemia-initiating cells in adult T-cell leukemia/lymphoma (ATL), arsenic-based treatment gave promising signs of efficacy in patients with disease that was refractory to standard treatment [6]. Schlaak *et al*. recently reported the case of a patient with metastatic melanoma refractory to two lines of chemotherapy. They successfully treated him with a combination of a cytotoxic agent and an anti-CD20 monoclonal humanized antibody to eradicate the sub-population of CD20-expressing cells with stemness characteristics in the melanoma metastases of this patient [7].

Although many questions remain, the results of our study support cancer stem-cell evaluation in biopsies of patients treated with sunitinib, and further research on the role of hypoxia in tumors resistant to anti-angiogenic drugs.
